# Benign cephalic histiocytosis: exuberant manifestation in an infant^☆^

**DOI:** 10.1016/j.abd.2022.11.008

**Published:** 2024-03-04

**Authors:** Ana Flávia Teixeira de Abreu, Rebecca Perez de Amorim, Pedro Marciano de Oliveira, Marcelo Padovani de Toledo Moraes, Silvio Alencar Marques

**Affiliations:** Department of Infectology, Dermatology, Imaging Diagnosis and Radiotherapy, Faculty of Medicine, Universidade Estadual Paulista, Botucatu, SP, Brazil

Dear Editor,

Histiocytoses are rare diseases resulting from the proliferation of cells derived from dendritic cells or macrophages. Clinical manifestations vary from benign lesions to severe, disseminated forms. The 1987 classification suggested the existence of three large groups: histiocytosis derived from Langerhans cells, non-Langerhans and malignant histiocytosis.^1^ However, a new, revised classification system consists of five groups of diseases. Group L: Langerhans histiocytosis, Group M: Malignant histiocytosis, Group R: Rosai-Dorfman disease and non-cutaneous non-Langerhans histiocytosis, Group H: Hemophagocytic lymphohistiocytosis and macrophage activation syndrome, and Group C which includes non-Langerhans histiocytosis located in the skin and mucous membranes, including benign cephalic histiocytosis (BCH).^2^ BCH is rare, occurs in the first three years of life, and is self-resolving, with lesions appearing mainly in the cephalic segment. The case reported herein describes a female infant, aged one month and 21 days, presenting rapidly evolving lesions for ten days. On examination, the patient was in good condition, with isolated or confluent erythematous-violaceous macules and papules, particularly affecting the upper third of the face and isolated lesions on the cervical and trunk regions, and absence of acral or mucosal lesions (Fig. 1). The initial hypotheses were benign cephalic histiocytosis or Hashimoto-Pritzker histiocytosis (HPH). Histopathology showed a diffuse histiocytic infiltrate in the superficial papillary dermis up to the middle portion of the reticular dermis of cells with an oval nucleus and clear cytoplasm (Fig. 2). Immunostaining was positive for fascin and CD68 and negative for CD1a and S100, characterizing a non-Langerhans histiocytosis (Fig. 3). Head, lung and abdominal computed tomographic investigation was negative, confirming the diagnosis of BCH. The approach was to reassure the parents, prescribing hydrocortisone and periodic monitoring.

**Figure 1:**
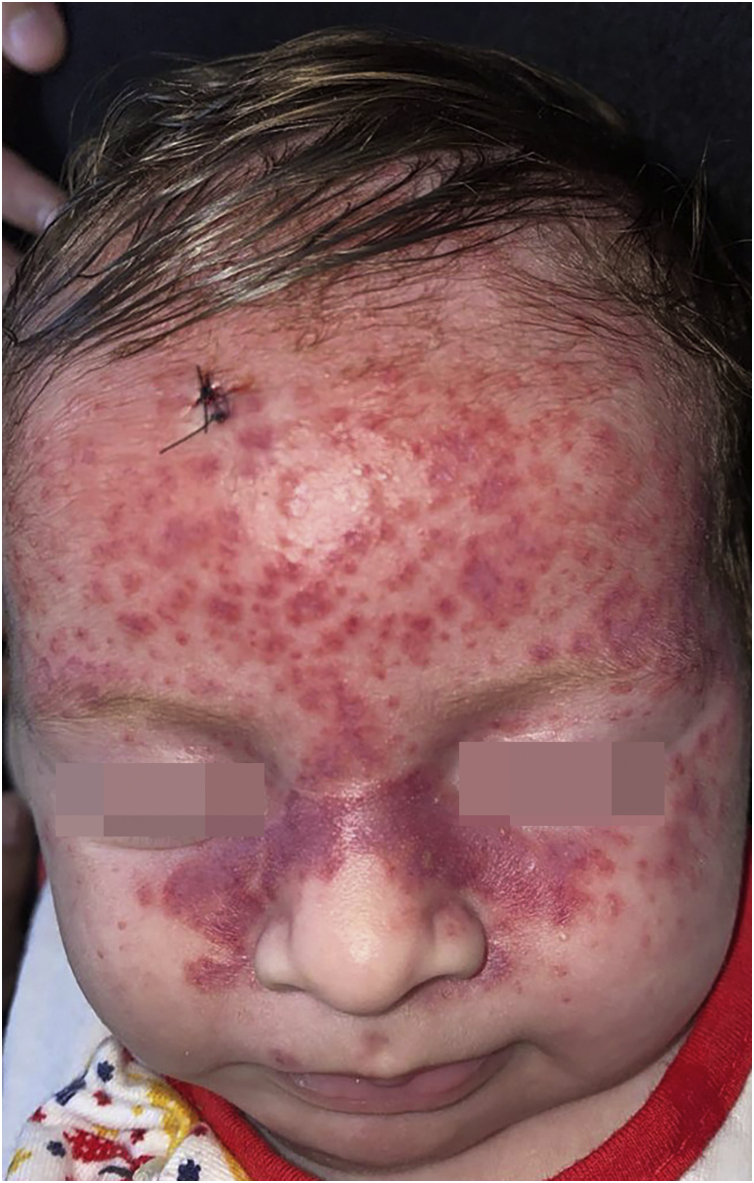
Numerous erythematous-violaceous macules, papules, isolated or confluent, particularly affecting the upper third of the face.

**Figure 2:**
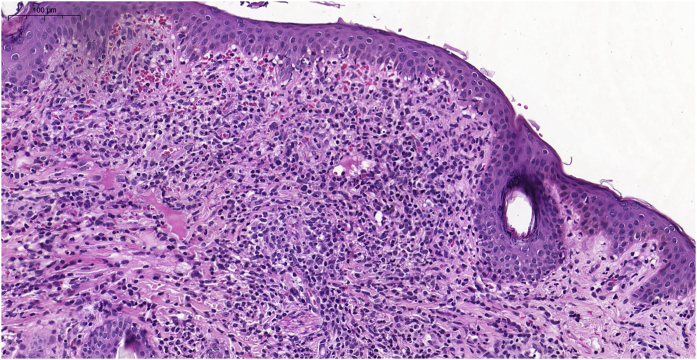
Histopathological examination showing a diffuse histiocytic infiltrate, in the superficial dermis up to the middle portion of the reticular dermis.

**Figure 3:**
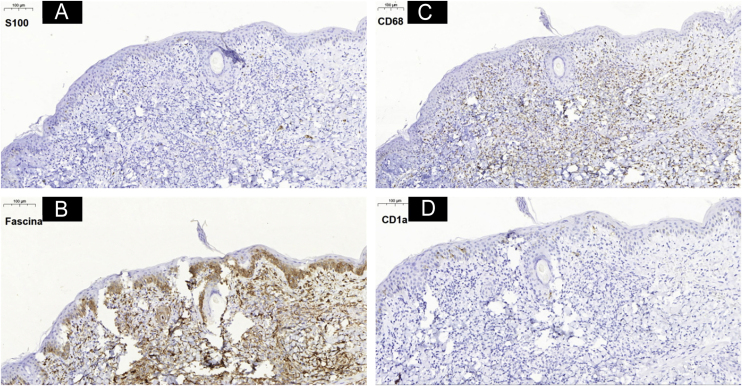
Immunohistochemistry analysis showing positivity for markers (3B) fascin and (3C) CD68 and negativity for (3A) S100 and (3D) CD1a.

Patient evolution showed an increase in lesions and expansion of the affected area. However, on the 50th day of follow-up, the lesions appeared to be resolving and on the 60th day there was practically complete resolution (Fig. 4).

**Figure 4:**
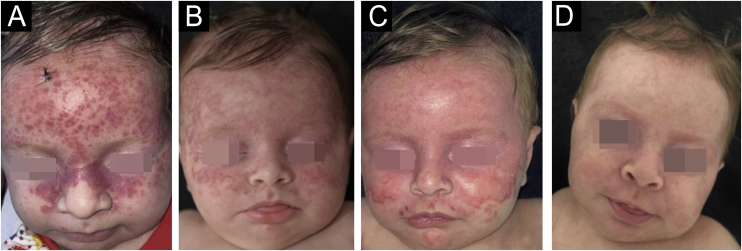
(A) Infiltrated reddish macules and papules, mainly on the face, without involvement of acral areas, mucous membranes or internal organs. (B‒D) Evolution of the lesions during the follow-up, on the 22nd, 36th and 50th days, respectively.

BCH was described by Gianotti et al. in 1971^3^ and an estimated 70 cases were described in the English-language literature by 2017.^4^ The lesions appear around the 2nd to the 6th month of life and are located on the face and cervical region, rarely affecting the scalp. It can also affect the trunk and roots of the limbs.^5^ They have an erythematous, or erythematous-violaceous color, are either macules or papules, and may converge and increase in number and area until stabilizing. Spontaneous regression can occur up to the 50th month of age.

The differential diagnosis, in addition to HPH, is made with juvenile xanthogranuloma (JXG) and mastocytosis. The clinical aspects and negative immunostaining for CD1a and S100 ruled out HPH. The absence of yellow-orange color, lesions practically restricted to the face and histopathological absence of Touton giant cells ruled out JXG. Mastocytosis, a more distant hypothesis, was excluded due to the clinical appearance, absence of Darier sign and absence of mast cell aggregates on histopathology. There are isolated reports of an association between BCH and JXG and even the appearance of diabetes insipidus in two patients, at some time after dermatological resolution.^2^, ^4^ Therefore, long-term monitoring must be maintained.

## Financial support

None declared.

## Authors’ contributions

Ana Flávia Teixeira de Abreu: Design and planning of the study; drafting and editing of the manuscript; collection, analysis and interpretation of data; effective participation in research orientation; intellectual participation in the propaedeutic and/or therapeutic conduct of the studied cases; critical review of the literature; critical review of the manuscript; approval of the final version of the manuscript.

Rebecca Perez de Amorim: Drafting and editing of the manuscript; collection, analysis and interpretation of data; intellectual participation in the propaedeutic and/or therapeutic conduct of the studied cases; critical review of the literature; critical review of the manuscript.

Pedro Marciano de Oliveira: Drafting and editing of the manuscript; collection, analysis and interpretation of data; intellectual participation in the propaedeutic and/or therapeutic conduct of the studied cases; critical review of the literature; critical review of the manuscript.

Marcelo Padovani de Toledo Moraes: Drafting and editing of the manuscript; collection, analysis and interpretation of data; intellectual participation in the propaedeutic and/or therapeutic conduct of the studied cases; critical review of the literature; critical review of the manuscript.

Silvio Alencar Marques: Design and planning of the study; drafting and editing of the manuscript; effective participation in research orientation; intellectual participation in the propaedeutic and/or therapeutic conduct of the studied cases; critical review of the literature; critical review of the manuscript; approval of the final version of the manuscript.

## Conflicts of interest

None declared.
